# Investigation of the antimicrobial, antioxidant, hemolytic, and thrombolytic activities of *Camellia sinensis*, *Thymus vulgaris*, and *Zanthoxylum armatum* ethanolic and methanolic extracts

**DOI:** 10.1002/fsn3.3569

**Published:** 2023-07-24

**Authors:** Sobia Rafique, Mian Anjum Murtaza, Iram Hafiz, Kashif Ameer, Mir Muhammad Nasir Qayyum, Shazia Yaqub, Isam A. Mohamed Ahmed

**Affiliations:** ^1^ Institute of Food Science and Nutrition University of Sargodha Sargodha Pakistan; ^2^ Institute of Chemistry University of Sargodha Sargodha Pakistan; ^3^ Department of Agriculture and Food Technology Karakoram International University Gilgit Pakistan; ^4^ Punjab Food Authority Lahore Pakistan; ^5^ Department of Food Science and Nutrition, College of Food and Agricultural Sciences King Saud University Riyadh Saudi Arabia; ^6^ Faculty of Agriculture, Department of Food Science and Technology University of Khartoum Shambat Sudan

**Keywords:** antioxidant activities, green tea, microbial assays, Tejphal, thyme, toxicological assays

## Abstract

*Camellia sinensis* is rich in antioxidants such as polyphenols; *Thymus vulgaris* contains bioactive compounds (flavonoids, terpenoids, and tannins) and *Zanthoxylum armatum* is primarily composed of volatile oils, amides, alkaloids, flavonoids, lignan, and coumarin. The antibacterial, antifungal, biofilm inhibition, antioxidant, hemolytic, and thrombolytic activities of *Camellia sinensis*, *Thymus vulgaris*, and *Zanthoxylum armatum* ethanol and methanol extracts at different concentrations (30%, 50%, and 80%) were determined. The antioxidant activity and content were measured as free radical scavenging assay (DPPH), total flavonoid content (TFC), and total phenolic content (TPC). Furthermore, hemolytic and thrombolytic analysis was carried out to determine toxicity. In antimicrobial assays, 80% methanol thyme extract showed highest (15.31 mm) antibacterial activity against *Bacillus subtilis*, and 80% ethanol green tea extract showed optimal antibacterial activity against *Staphylococcus aureus*. Ethanol 30% green tea extract resulted in highest (26.61 mm) antifungal activity against *Aspergillus niger*. The maximum (54.73%) biofilm inhibition was resulted by methanol 50% thyme extract for *Escherichia coli*. In antioxidant activity and content, methanol 50% green tea extract had highest (80.82%) antioxidant activity, whereas, ethanol 80% green tea extract had maximum (1474.55 mg CE/g DW) TFC and methanol 80% green tea extract had maximum (593.05 mg GAE/g) TPC. In toxicological assays, methanol 30% green tea extract had highest (25.28%) thrombolytic activity, and ethanol 80% tejphal extract had maximum (18.24%) hemolytic activity. This study has highlighted the significant antimicrobial, antioxidant, hemolytic, and thrombolytic activities of *Camellia sinensis*, *Thymus vulgaris*, and *Zanthoxylum armatum* extracts that could be beneficial to treat various diseases (cancer, diabetes, and respiratory diseases) and may be utilized as functional ingredient in the preparation of functional foods and drinks.

## Graphical abstract

The antibacterial, antifungal, biofilm inhibition, antioxidant, hemolytic and thrombolytic activities of *Camellia sinensis, Thymus vulgaris* and *Zanthoxylum armatum* ethanol and methanol extracts at different concentrations were determined. This study has highlighted the significant antimicrobial, antioxidant, hemolytic and thrombolytic activities of *Camellia sinensis*, *Thymus vulgaris* and *Zanthoxylum armatum* extracts that could be beneficial to treat various diseases (cancer, diabetes and respiratory diseases) and may be utilized as functional ingredient in preparation of functional foods and drinks.

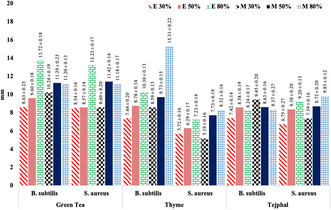

## 1 INTRODUCTION

For decades, natural ingredients have obtained increasing attention owing to their health benefits to formulate medicines for reducing incidence of health issues (Ameer, Shahbaz, & Kwon, 2017; Poveda et al., 2018). The significance of medicinal plants is due to their high diversity of highly valuable molecules, such as vitamins, phenolic compounds, tocopherols, and carotenoids (Feng et al., 2022). Nowadays, bioactive compounds are in high demand because of their antioxidant properties and their ability to fight many chronic diseases, such as obesity, diabetes, and cancer (Jiang et al., 2022; Jiang, Feng, et al., 2021; Johnson et al., 2023).

Medicinal plants exhibit antimicrobial potential, which is generally credited to the presence of polyphenols in them. The antioxidant potential of plant‐derived compounds is attributed to the existence of high concentrations of phenolic compounds, which have strong H‐donating activity (Huo et al., 2022; Jiang, Ramachandraiah, et al., 2021), and other bioactive compounds, such as carotenoids, flavonoids, phenolic diterpenes, and anthocyanidins (Hong et al., 2022). Plant extracts have generally recognized as safe (GRAS) status for intended consumers (Ameer et al., 2022).

Green tea is considered as commonly used beverage with functional properties worldwide because of the high amount of antioxidants in its chemical composition (Raza et al., 2022). The presence of antioxidant compounds in green tea includes flavanols, phenolic acids, and flavonoids, which have proven health benefits (Zhao, Ameer & Eun, 2021). The consumption of green tea is associated with a reduced risk of cancer and circulatory system diseases due to its anti‐inflammatory, antioxidant, and antiviral functions, which stimulate detoxification and the immunological process (Zhao, Lu & Ameer, 2021).

Thyme (*Thymus vulgaris*) and its extracts has antispasmodic, antioxidant, antiseptic, antimicrobial, antifungal, antiviral, and antitussive properties, due to which they have been used in traditional medicine for the treatment of certain respiratory diseases like bronchitis, asthma, and other pathologies (Oji et al., 2020). The occurrence of several bioactive compounds has been confirmed by the phytochemical screening of thyme extracts. These bioactive compounds include terpenoids, tannins, and flavonoids, which have antitumor, antimicrobial, hypoglycemic, hematological, and antioxidant properties (Irfan et al., 2023).

Tejphal (*Zanthoxylum armatum*) exhibits strong antimicrobial potential. The highest inhibition zone was revealed against *Bacillus subtilis* (Bhatt et al., 2018). Fruits, stems, roots, and leaves of tejphal are used in biomedicine as raw materials due to their antioxidant, anti‐inflammatory, antitumor, antibacterial, and analgesic effects (Iftikhar et al., 2023; Irfan et al., 2022). The key chemical components of tejphal are coumarin, volatile oils, flavonoids, alkaloids, lignans, and amides. This plant exhibits free radical activity as a good source of antioxidants (Bhatt et al., 2018).

Worldwide, there are numerous studies being conducted on the antioxidant potential of the bioactive components from both ordinary and nontraditional plants (Ameer et al., 2017; Ameer, Chun, & Kwon, 2017). However, there are very few reports available on the investigation of the therapeutic potentials of *Camellia sinensis*, *Thymus vulgaris*, and *Zanthoxylum armatum* extracts in ethanol and methanol at different concentrations (30%, 50%, and 80%). Hence, the current study was conducted to investigate the antimicrobial, antioxidant, and toxicological assays of *Camellia sinensis*, *Thymus vulgaris*, and *Zanthoxylum armatum* extracts in different concentrations of a couple of organic solvents.

## 2 MATERIALS AND METHODS

### 2.1 Sample collection

Green tea (*Camellia sinensis*) leaves, Thyme (*Thymus vulgaris* L.) leaves, and Tejphal (*Zanthoxylum armatum*) fruits were purchased from local market of Sargodha, Punjab, Pakistan.

### 2.2 Extraction of bioactive compounds

Sonication technique was employed to extract bioactive compounds from green tea and thyme leaves and tejphal fruit powder using extractants, such as ethanol and methanol at different concentrations of 30%, 50%, and 80%. For sonication, sonicator (Bandelin RK 510H Sonorex, Heinrichstrabe, Berlin, Germany) was used for 4 min. The solvent‐free extract was obtained using rotary evaporator (Buchi Labortechnik, Rotavapor R‐300, Meierseggstrasse, Flawil, Switzerland) at 60°C till ethanol and methanol solvent evaporated and stored at –4°C till further analyses (Ameer et al., 2022).

### 2.3 Antimicrobial activity

Antibacterial activity was measured by disk diffusion method and results were expressed in terms of mm by following the method of Ameer et al. (2020). Well‐diffusion method was employed to determine antifungal activity of plant extracts and results were expressed in terms of mm. The biofilm inhibition of studied plant extracts was measured by spectrophotometric assay at 570 nm and results were expressed as percentage (%) by following the method of Regev‐Shoshani et al. (2010). Inhibition percentage was calculated by using the following formula given in Equation 1:

(1) $$\text{Inhibition}\;\left(\%\right)=\frac{\mathrm{OD}\;\left(\text{control}\right)-\mathrm{OD}\;\left(\text{samples}\right)}{\mathrm{OD}\;\left(\text{control}\right)}\times 100$$

### 2.4 DPPH radical scavenging activity

The antioxidant capacity of plant extracts was measured using 2, 2‐diphenyl‐1‐1 picrylhydrazyl (DPPH) radical‐scavenging activity according to the method of Maeng et al. (2017) with slight modifications. Methanol extract (0.5 mL) of the sample at various concentrations was added to 2.5 mL of freshly prepared DPPH solution (25 mg/L). The mixture was incubated for 30 min at room temperature and the decrease in absorbance at the end of incubation period was measured at 515 nm by a spectrophotometer (Shimadzu, Japan). Pure methanol was used as blank. BHA and α‐tocopherol were employed as reference standards for this assay. The percent DPPH scavenged by each sample was calculated by the following equation:

(2) $$\%\text{DPPH scavenging activity}\;=\;\frac{A0–A1}{A0}\times 100$$

### 2.5 Determination of total phenolic and flavonoid contents

Aluminum chloride (AlCl_3_) colorimetric method was used for flavonoid determination according to the method of Wu et al. (2021). Each extract (0.5 mL of 1:10 g/mL) in methanol was separately mixed with 1.5 mL of methanol, 0.1 mL of 10% AlCl_3_, 0.1 mL of 1 M potassium acetate, and 2.8 mL of distilled water. Then, stay time of about 30 min was given to reaction mixture at room temperature. The absorbance of the reaction mixture was measured at 415 nm using ultraviolet–visible spectrophotometer. The calibration curve was prepared by comparing with standard curve of quercetin solutions at concentrations ranging from 12.5 to 100 μg/mL in methanol.

A modified Folin–Ciocalteu method as described by Chang et al. (2002) was used for the determination of total phenolic contents. Each sample in an amount of 0.4 mL was mixed with 2 mL of the Folin–Ciocalteu reagent (diluted 10 times) and the reaction was mixed with 1.6 mL of 7.5% sodium carbonate. After 30‐min incubation at room temperature (28 ± 1°C), the absorbance was read at 750 nm using a ultraviolet–visible spectrophotometer (Shimadzu, Japan). The standard curve was prepared using gallic acid standard solutions of known concentrations, and the results were expressed as milligram of gallic acid equivalent per gram of sample on dry weight basis.

### 2.6 Hemolytic activity

The method of Jiang et al. (2020) with some modifications was followed to evaluate the ethanolic and methanolic extracts of selected medicinal plants on human erythrocytes (O blood groups). Healthy volunteers were selected to obtain human blood samples. The centrifugation of blood sample was carried out for 5 min at 5000 rpm. For hemolytic study, saline phosphate buffer was used to prepare 2% erythrocyte suspension.

In vitro conditions were followed to test the hemolytic activity of crude extracts of each selected medicinal plant. Different concentrations of extracts ranging between 50 and 500 μg/mL were poured into NaCl solution (0.85%) and mixed. Next, 2% suspension of human erythrocytes was added to the mixture and incubated at room temperature for 30 min. After which, centrifugation of the sample was performed and then absorbance of liberated hemoglobin was measured at 540 nm by using supernatant. Hemolysis percentage was measured using the following formula:

(3) $$\text{Hemolysis percentage}=\frac{\text{Sample absorbance}}{\text{Positive control absorbance}}\times 100$$

### 2.7 Thrombolytic activity

The method of Uddin et al. (2021) was followed to investigate the thrombolytic activity of the ethanolic and methanolic extracts of selected medicinal plants. Healthy volunteers were selected to draw 2.5 mL of venous blood and immediately collected in sterile preweighed microcentrifuge tube. Next, tubes containing blood samples were incubated for 45 min at 37°C. When the clot was formed, entire fluid was removed from each tube, and clot weight was determined by the following formula:

(4) $$\text{Weight of Clot}=\left(\mathrm{Wt}.\;\text{of tube containing clot}–\mathrm{Wt}.\;\text{of empty tube}\right)$$

For positive control, streptokinase (SK) vial and PBS (2.5 mL), while for negative control, 100 μL of distilled water was separately added along with 100 μL of selected medicinal plants extracts to the microcentrifuge tubes containing clots, then incubation of all tubes was done for 90 min at 37°C, and then clot lysis was observed in them. The serum released from samples was removed. For observing the difference in weight, tubes were again weighed after disruption of clot. Lastly, the percentage of clot lysis was calculated using the following formula:

(5) $$Cl\mathrm{ot}\;\text{lysis}\;\left(\%\right)=\frac{\mathrm{Wt}.\;\text{of released clot}}{\mathrm{Wt}.\;\text{of clot}}\times 100$$

### 2.8 Statistical analysis

Results were statistically analyzed by using one‐way analysis of variance (ANOVA). Statistical differences were analyzed using paired *t*‐test. *p* < .05 was considered statistically significant. All values are expressed as mean ± SEM for three replicates (*n* = 3).

## 3 RESULTS AND DISCUSSIONS

### 3.1 Antibacterial activity

**FIGURE 1:**
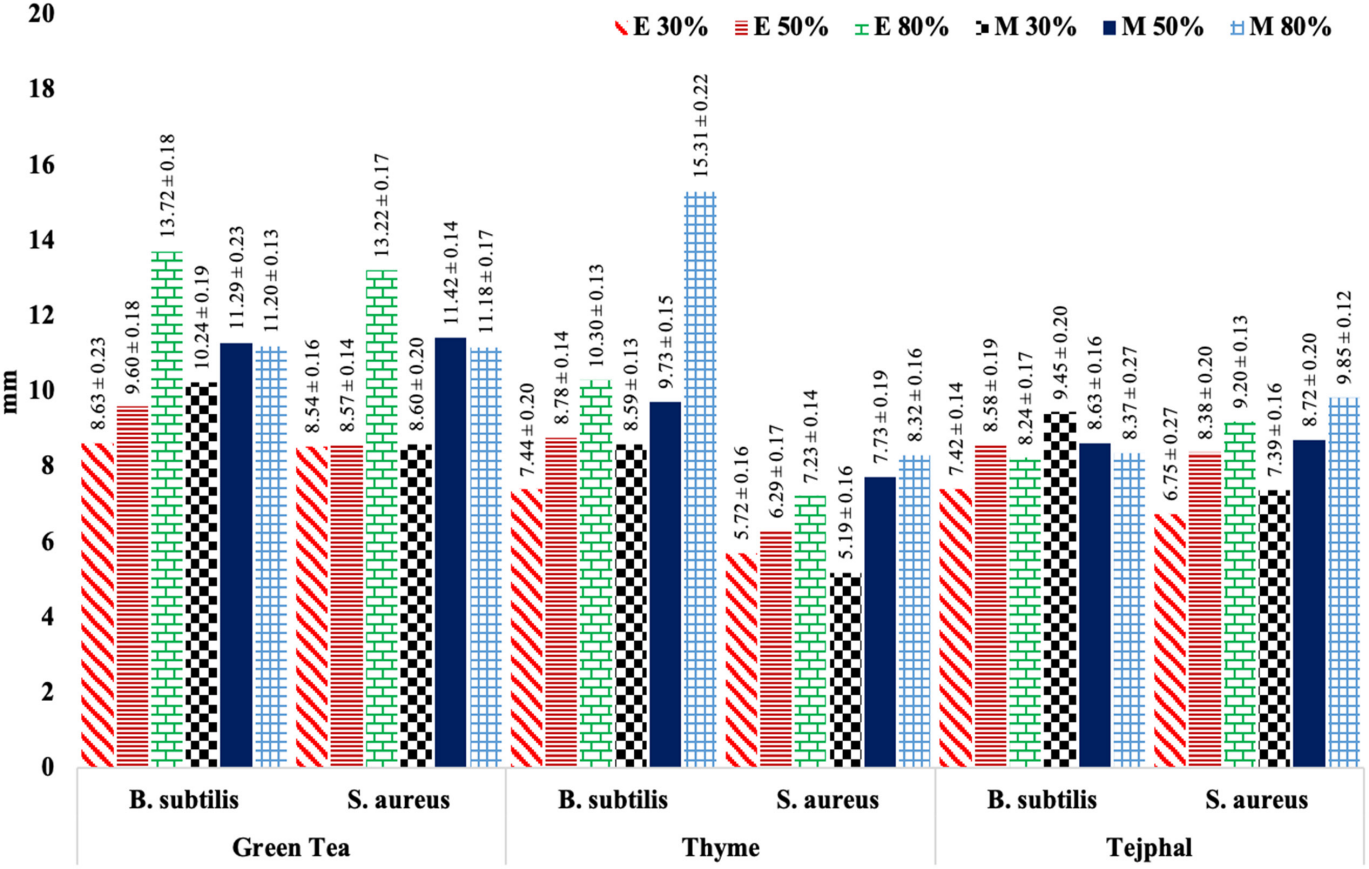
Antibacterial activity (mm) of selected medicinal plant extracts against *B. Subtilis* and *S. aureus*.

Results regarding antibacterial activity of green tea leaves, thyme leaves, and tejphal against *B. subtilis* and *S. aureus* are shown in Figure 1. It is clear from the data that plants' extracts have significant (*p* < .05) antibacterial activity against different pathogenic bacteria.

Green tea ethanolic extract showed maximum antibacterial activity against *B. Subtilis* (13.72 mm in 80% ethanol conc.); however, other solvent concentrations were least effective against *B. subtilis* and *S. Aureus*. Antioxidants of green tea (polyphenols and catechins) have major influence on inhibiting bacterial growth and development. The catechins include epigallocatechin (EGC), epicatechin (EC), gallocatechin gallate (GCG), and epicatechin gallate (ECG) which are responsible for green tea antibacterial potential (Pelczar & Chan, 2005). Tea extracts have very selective antibacterial activity. This antibacterial activity difference depends upon several factors, such as concentration and type of extracts and bacterial species (either growth stimulatory or inhibitory) (Zhao et al., 2019). Constituents of tea also exhibit antimutagenic, antibacterial, anticarcinogenic, and antiviral properties (Toda et al., 1991). Kumar et al. (2012) in their study investigated the antibacterial activity of green tea extracts at 10, 20, and 30 μL concentrations of ethanol, methanol, and water, respectively. It was evident that methanolic extracts had maximum antibacterial activity against isolated bacteria: *Staphylococcus*, *Streptococci*, *Bacillus*, *Pseudomonas*, and *Proteus* as compared to ethanolic and aqueous extracts.

For thyme, greater zone of inhibition was observed in case of methanolic extract against *B. subtilis* (15.31 mm in 80% methanol conc.); however, other ethanolic and methanolic concentrations showed the least effectiveness against *B. subtilis* and *S. Aureus*. Borugă et al. (2014) reported that thyme's antibacterial activity primarily depends upon the thymol (phenolic compound) and terpene hydrocarbons. Šojić et al. (2020) conducted a study on thyme plant extract and reported that carvacrol, thymol, γ‐terpinene and cymene are the most active constituents of thyme with a wide spectrum of antimicrobial property. Yang et al. (2008) reported that thyme's antibacterial activity could be attributed to its main constituents, which are well known to possess significant antibacterial activity including β‐pinene, α‐terpineol, α‐terpinolene, caryophyllene, limonene, γ‐terpinene, cymene, and bornyl acetate.

Similarly, tejphal methanolic extract showed maximum activity against *S. aureus* (9.85 mm in 80% methanol conc.) as compared to other concentrations. Phuyal et al. (2019) reported that antibacterial activity of tejphal was associated with its active constituents such as alkaloids, terpenoids, sterols, flavonoids, and coumarins. Phuyal et al. (2020) also reported that the antibacterial activity of *Z. armatum* was attributed to the higher phenolic and flavonoid contents present in the extract. The phytochemical activity of numerous phytoconstituents present in plant matrix may be responsible for the antibacterial activities of extracts. According to reports, *Z. armatum* has a wide range of antibacterial compounds in its composition with different chemical structures, such as terpenoids, flavonoids, coumarins, sterols, and alkaloids (Manandhar et al., 2019).

### 3.2 Antifungal activity

Results regarding antifungal activity of green tea leaves, thyme leaves, and tejphal against *Aspergillus niger* are shown in Table 1. It can be easily understood from the data that studied plants' extracts exhibited significantly (*p* < .05) high antifungal activity against *Aspergillus niger*.

**TABLE 1:** Antifungal activity (mm) of selected medicinal plant extracts against *A. niger*.

Concentrations (%)	Green tea	Thyme	Tejphal
Ethanol 30	26.61 ± 1.14^a^	14.98 ± 0.29^b^	8.26 ± 0.18^c^
Ethanol 50	18.95 ± 1.34^b^	18.04 ± 1.45^a^	13.65 ± 0.28^a^
Ethanol 80	12.95 ± 1.62^d^	14.11 ± 1.52^b^	11.95 ± 0.12^b^
Methanol 30	14.13 ± 1.39^c^	17.65 ± 0.51^a^	9.06 ± 0.07^c^
Methanol 50	13.82 ± 1.35^c^	12.82 ± 1.17^d^	7.12 ± 0.08^d^
Methanol 80	17.95 ± 0.94^b^	16.88 ± 0.23^c^	8.31 ± 0.18^c^

*Note*: Mean values are the result of three replications (*n* = 3) and are shown as mean ± standard deviation (S.D.). Means carrying the same small letters (a–d) in the column are significantly (*p* < .05) different from each other.

For green tea, results showed maximum antifungal activity (inhibition zone: 26.61 mm in 30% ethanol conc.) against *Aspergillus niger*. Green tea exhibits strong and diverse range of antifungal compounds against Candidiasis caused by Candida fungus. Probably, this characteristic was attributed to the presence of catechin, epigallocatechin gallate (EGCG), and epigallocatechin (EGC) in teas (Hirasawa & Takada, 2004). Sitheeque et al. (2009) in their study reported the green and black tea catechins' antifungal activity against *Candida albicans*.

For thyme, results showed maximum antifungal activity (inhibition zone: 18.04 mm in 50% ethanol conc.) against *Aspergillus niger*. Rao and Singh (1994) in their study compared the effects of natural and synthetic fungicides and reported that thyme fruit extract had strong antifungal activity against *Ceratocystis paradoxa* and *Colletotrichum falcatum* (sugarcane pathogens) due to the presence of geraniol even was comparable to that with antifungal activity of commercial synthetic fungicides. In another study carried out by Bafi‐Yeboa et al. (2005), the authors evaluated antifungal activity of root, bark, leaves, stem, and fruit of thyme using 11 strains of fungi. For tejphal, results showed maximum antifungal activity (inhibition zone: 13.65 mm in 50% ethanol concentration) against *Aspergillus niger*. Studies reported that antifungal activity of tejphal may possibly be associated with alkaloids (Liu et al., 2020).

### 3.3 Biofilm formation inhibition activity

Results regarding biofilm formation inhibition activity of green tea leaves, thyme leaves, and tejphal fruit extracts against *E. coli* are given in Table 2. For green tea, results showed significantly highest (*p* < .05) biofilm formation inhibition activity (47.75% in 80% ethanol) against *E. coli*. The tea catechins have been reported to retard the growth and the ability to form and maintain biofilms by *C. albicans* (Nam et al., 2001). Cho et al. (2007) in their study reported that high concentrations of green tea extracts caused toxicity in *E. coli* by disrupting membrane components that led to death of cells and inhibited biofilm formation. Nataro (2006) reported that catechin, epigallocatechin, and epicatechin gallate in green tea leaves inhibited the biofilm formation because of *E. coli*. It is already reported in published literature that green tea exhibits antibiofilm properties. The green tea polyphenols, also known as catechins, are primarily responsible for their health‐promoting effects. Epigallocatechin‐3‐gallate (EGCG), epigallocatechin, epicatechin‐3‐galate, and epicatechin are the four primary catechin types.

**TABLE 2:** Biofilm formation inhibition activity (%) of selected medicinal plant extracts for *E. coli*.

Concentration (%)	Green tea	Thyme	Tejphal
Ethanol 30	46.93 ± 1.59^a^	27.32 ± 0.68^d^	3.53 ± 0.23^e^
Ethanol 50	45.90 ± 1.14^a^	33.93 ± 1.91^c^	14.70 ± 1.14^d^
Ethanol 80	47.75 ± 1.42^a^	24.40 ± 0.47^e^	46.05 ± 1.66^a^
Methanol 30	35.59 ± 0.54^b^	44.89 ± 1.35^b^	42.63 ± 1.12^b^
Methanol 50	41.69 ± 1.12^c^	54.73 ± 1.80^a^	37.44 ± 1.34^c^
Methanol 80	2.27 ± 0.23^d^	43.13 ± 0.86^b^	46.14 ± 0.41^a^

*Note*: Mean values are the result of three replications (*n* = 3) and are shown as mean ± standard deviation (S.D.). Means carrying the same small letters (a–d) in the column are significantly (*p* < .05) different from each other.

For thyme, results showed the highest biofilm formation inhibition activity (54.73% in 50% methanol concentration) against *E. coli*. Sandasi et al. (2008) reported that thyme extract and its essential oil can inhibit the formation of biofilms from *C. albicans*, *L. monocytogense*, *and E. coli*. Alibi et al. (2020) also reported biofilm formation inhibition properties of *Thymus vulgaris*. For tejphal, results showed highest biofilm formation inhibition activity (46.14% in 80% ethanol conc.) against *E. coli*.

### 3.4 DPPH radical scavenging activity

Studied plant extracts were assessed for their antioxidant activities by using DPPH radical scavenging activity (DPPH‐RSA) assay. Methanolic extracts (50%) of green tea, thyme, and tejphal showed DPPH‐RSA of 80.82%, 76.72%, and 71.07%, respectively, as mentioned in Table 4.

Khalaf et al. (2008) carried out a study on ethanolic and methanolic green tea extracts through DPPH assay, and they reported that it had higher DPPH‐RSA. According to Gholivand et al. (2010), the antioxidant activity of thyme extracts directly depends upon the high phenolic content which acts as scavenger of the free radicals. Similarly in another study, Mohammed et al. (2017) reported that the thymol and carvacrol (phenolic compounds) present in thyme were responsible for DPPH‐RSA, while DPPH‐RSA of tejphal was attributed to the high content of linalool, β‐ocimene, camphene, γ‐terpinene, α‐copaene, cymene, and bornyl acetate (Dongmo et al., 2008).

The RSA of *Z. armatum* extracts may be attributed to the presence of polyphenols, flavonoids, and phenolic compounds and polyphenols are usually responsible for the majority of the antioxidant activity of plants (Nooreen et al., 2017). Antioxidants are absolutely crucial substances that have the potential to safeguard the body from free radical‐induced oxidative stress. Because of the hydrogen‐donating property of their hydroxyl groups, plant polyphenols act as reducing agents and antioxidants (Aberoumand & Deokule, 2008).

### 3.5 Total flavonoid content (TFC) and total phenolic content (TPC)

Results regarding total flavonoid and total phenolic contents of green tea leaves, thyme leaves, and tejphal fruit extracts against *E. coli* are given in Table 3.

**TABLE 3:** Total phenolic (mg GAE/g DW basis) and flavonoid contents (mg CE/g DW basis) of selected medicinal plant extracts.

Concentration (%)	Green tea		Thyme		Tejphal	
	TPC	TFC	TPC	TFC	TPC	TFC
Ethanol 30	362.05 ± 2.74^b^	1382.23 ± 15.33^b^	109.90 ± 1.48^b^	154.10 ± 2.49^c^	41.77 ± 1.79^e^	92.76 ± 1.90^d^
Ethanol 50	184.85 ± 1.74^d^	1413.23 ± 10.33^a^	106.12 ± 0.49^b^	158.72 ± 4.26^c^	105.92 ± 1.54^b^	187.49 ± 2.60^b^
Ethanol 80	380.92 ± 1.34^b^	1474.55 ± 7.33^a^	131.75 ± 1.90^a^	256.89 ± 2.33^a^	132.26 ± 2.35^a^	203.26 ± 3.01^a^
Methanol 30	292.22 ± 1.52^c^	981.17 ± 11.50^d^	39.53 ± 1.53^d^	92.96 ± 2.19^d^	50.88 ± 1.40^d^	124.08 ± 1.52^c^
Methanol 50	189.99 ± 1.60^d^	1121.95 ± 3.24^c^	62.78 ± 1.14^c^	104.08 ± 2.05^d^	73.58 ± 2.05^c^	126.93 ± 2.04^c^
Methanol 80	593.05 ± 2.31^a^	1171.30 ± 7.34^c^	33.01 ± 1.59^d^	189.88 ± 1.29^b^	73.71 ± 1.63^c^	136.45 ± 2.34^c^

*Note*: Mean values are the result of three replications (*n* = 3) and are shown as mean ± standard deviation (S.D.). Means carrying the same small letters (a–e) in the column are significantly (*p* < .05) different from each other.

**TABLE 4:** DPPH radical scavenging activity (%) of selected medicinal plant extracts.

Concentration (%)	Green tea	Thyme	Tejphal
Ethanol 30	76.59 ± 1.36^b^	66.94 ± 2.00^b^	50.82 ± 1.18^c^
Ethanol 50	77.15 ± 0.74^b^	70.60 ± 1.23^a^	53.57 ± 2.00^c^
Ethanol 80	77.70 ± 1.92^b^	75.87 ± 1.27^a^	56.86 ± 1.31^c^
Methanol 30	75.73 ± 1.57^b^	64.74 ± 1.61^b^	39.34 ± 0.55^d^
Methanol 50	80.82 ± 1.19^a^	76.72 ± 1.38^a^	71.07 ± 1.77^a^
Methanol 80	78.22 ± 1.49^a^	60.00 ± 1.32^c^	64.63 ± 1.99^b^

*Note*: Mean values are the result of three replications (*n* = 3) and are shown as mean ± standard deviation (S.D.). Means carrying the same small letters (a–d) in the column are significantly (*p* < .05) different from each other.

**TABLE 5:** Hemolytic activity (%) of selected medicinal plant extracts.

Concentration (%)	Green tea	Thyme	Tejphal
Ethanol 30	11.33 ± 0.10 ^b^	2.09 ± 0.05 ^a^	6.52 ± 0.20 ^c^
Ethanol 50	2.21 ± 0.09 ^c^	1.32 ± 0.07 ^b^	4.77 ± 0.13
Ethanol 80	2.79 ± 0.12 ^c^	1.48 ± 0.12 ^b^	18.24 ± 0.07 ^a^
Methanol 30	16.18 ± 0.11 ^a^	2.40 ± 0.14 ^a^	5.09 ± 0.03 ^d^
Methanol 50	3.70 ± 0.12 ^c^	1.59 ± 0.07 ^b^	5.68 ± 0.20 ^c^
Methanol 80	1.20 ± 0.04 ^d^	1.07 ± 0.04 ^c^	12.01 ± 0.02 ^b^

*Note*: Mean values are the result of three replications (*n* = 3) and are shown as mean ± standard deviation (S.D.). Means carrying the same small letters (a–d) in the column are significantly (*p* < .05) different from each other.

**TABLE 6:** Thrombolytic activity (% clot lysis) of selected medicinal plant extracts.

Concentrations (%)	Green tea	Thyme	Tejphal
Ethanol 30	5.84 ± 0.13^c^	23.18 ± 0.25^a^	4.20 ± 0.04^c^
Ethanol 50	2.66 ± 0.25^d^	14.65 ± 0.25^b^	8.19 ± 0.11^a^
Ethanol 80	21.82 ± 0.15^a^	6.39 ± 0.14^d^	6.16 ± 0.06^b^
Methanol 30	25.28 ± 0.13^a^	11.24 ± 0.16^c^	3.38 ± 0.07^d^
Methanol 50	16.31 ± 0.22^b^	6.61 ± 0.23^d^	3.18 ± 0.11^d^
Methanol 80	15.55 ± 0.05^b^	12.65 ± 0.27^b^	4.13 ± 0.05^c^

*Note*: Mean values are the result of three replications (*n* = 3) and are shown as mean + standard deviation (S.D.). Means carrying the same small letters (a–d) in the column are significantly (*p* < .05) different from each other.

Generally, phytoconstituents found in medicinal plants are polyphenols and flavonoids which exhibit several biological activities like antioxidant potential, etc. Green tea extract showed the highest (*p* < .05) total flavonoid content of 593.05 mg CE/g with 80% methanol and highest TPC was 1474.55 mg GAE/g in 80% ethanol. Lorenzo and Munekata (2016) also reported that the antioxidant activity of green tea depends upon the phenolic content and concluded that flavonoids exhibit the ability to scavenge free radicals including reactive oxygen species (Zou et al., 2016).

Thyme showed maximum total flavonoid content (131.75 mg CE/g) and total phenolic content (256.89 mg GAE/g) with 80% ethanol. Jabri‐Karoui et al. (2012) reported that thyme is a rich source of phenolic monoterpenes: thymol and carvacrol. Moreover, Mancini et al. (2015) reported that thyme extract has been recognized for its higher antioxidant capacity due to the presence of phenolic monoterpenes as well as sesquiterpenes. Köksal et al. (2017) carried out a preliminary phytochemical analysis of thyme's ethanolic extract and reported that antioxidant properties were due to phenolic and flavonoid contents.

Tejphal showed the maximum total flavonoid content (132.26 ± 2.35 mg CE/g) and total phenolic content (203.26 mg GAE/g) with 80% ethanol. Polyphenols and flavonoids in tejphal exhibit several biological activities such as anticancer, antimicrobial, antioxidant, and antiviral (Havsteen, 2002). Jung et al. (2003) reported that earlier findings have revealed the presence of phytochemicals such as phenolic acids, alkaloids, and flavonoids in tejphal. Saeed et al. (2004) carried out a study on ethanolic extracts of tejphal and reported that the antioxidant activity could be associated with high content of phenolics and flavonoids present in tejphal.

### 3.6 Hemolytic activity

Destruction of red blood cells is usually caused by lysis of membrane lipid bilayer also known as hemolysis which is related to potency and concentration of extract. Moreover, the hemolytic activity directly depends on the chemical composition of the extracts. Selected medicinal plants were assessed for cytotoxic activity through hemolytic assay to access the extracts' toxicity profile. Current research was carried out as in vitro hemolytic activity by using ethanolic and methanolic extracts at different concentrations and red cell suspension from volunteers (healthy human subjects). The mean values of hemolytic activity (%) of studied plant extracts are presented in Table 5. Resultantly among the studied plant extracts, green tea showed the highest hemolytic activity of 16.18% in 30% methanol, whereas thyme also exhibited maximum activity of 2.40% in 30% methanol. However, minimum activity (18.24%) was shown by tejphal in 80% ethanol.

Kunlawong et al. (2014) reported that the polyphenols present in green tea extracts protected erythrocyte oxidation and hemolysis of red blood cells. The antihemolytic activity exhibited by green tea was because of phytochemicals present in it as reported by Costa et al. (2009). Chaudhuri et al. (2007) reported that flavonoids present in thyme and other medicinal plants showed antihemolytic effects and positively affected the stability of erythrocyte membrane. Antihemolytic activity of thyme extract can be linked to its polyphenols and flavonoid content (de Freitas et al., 2008).

### 3.7 Thrombolytic activity

Studied plant extracts were evaluated for their thrombolytic activity and results of their mean values are given in Table 6. Among the studied plants, the highest (*p* < .05) thrombolytic activity showed by green tea was 25.28% in 30% methanol conc., thyme also exhibited the maximum activity of 23.18% with 30% ethanol conc. and the optimum activity was shown by the tejphal (8.19%) in 50% ethanol concentration.

Medicinal plant extracts possess thrombolytic activity that might be due to the presence of phytoconstituents, such as terpenoids, tannins, flavonoids, and alkaloids (Dwivedi, 2007). Atnasooriya et al. (2007) reported that green tea has potent thrombolytic activity owing to its ability to impair blood clotting in human as well as in animal subjects. Green tea is rich in phytochemicals, antioxidants, and flavonoids and had strong thrombolytic activity (Manukumar et al., 2013). Enomoto et al. (2001) in their study reported that thrombolytic activity of thyme was because of carvacrol that is also known as monoterpenoid phenol in published literature.

## 4 CONCLUSION

Green tea, thyme, and tejphal are excellent sources of bioactive compounds and possess strong antibacterial, antifungal, antiviral, and antioxidant activities. Briefly, this study has highlighted that 80% methanolic thyme extract showed maximum antibacterial activity, whereas 30% ethanolic green tea extract showed maximum antifungal activity. Moreover, 50% methanolic thyme extract showed maximum biofilm inhibition activity as compared to other extracts at different solvent concentrations. While 50% methanolic green tea extract exhibited maximum DPPH‐RSA, 80% ethanolic green tea extract showed maximum TFC. Methanolic 80% green tea extract showed maximum TPC. Whereas methanolic 30% green tea extract showed maximum thrombolytic activity and ethanolic 80% tejphal extract showed the highest hemolytic activity. It was concluded that *Camellia sinensis*, *Thymus vulgaris*, and *Zanthoxylum armatum* being a potential source of antimicrobial, antioxidant compounds, could be utilized as a proven therapeutic agent to manage and cure various diseases when used in functional foods and drinks.

## AUTHOR CONTRIBUTIONS

**Sobia Rafique:** Conceptualization (equal); data curation (equal); formal analysis (equal); investigation (equal); methodology (equal); visualization (equal). **Mian Anjum Murtaza:** Investigation (equal); methodology (equal); visualization (equal). **Iram Hafiz:** Supervision (equal); validation (equal). **Kashif Ameer:** Resources (equal); validation (equal); visualization (equal); writing – review and editing (equal). **Mir Muhammad Nasir Qayyum:** Validation (equal); visualization (equal); writing – review and editing (equal). **Shazia Yaqub:** Validation (equal); visualization (equal); writing – review and editing (equal). **Isam A. Mohamed Ahmed:** Resources (equal); validation (equal); writing – review and editing (equal).

## CONFLICT OF INTEREST STATEMENT

The authors declare that they have no conflict of interest.

## Data Availability

The data supporting the conclusions of this article are included in the manuscript.
